# Primary ectopic breast carcinoma: a case report

**DOI:** 10.1186/s13256-022-03670-7

**Published:** 2022-11-26

**Authors:** Leila Achouri, Amani Jellali, Houda Henchiri, Sabrine Boukhris, Yosra Zaaimi, Houyem Mansouri, Najet Mahjoub

**Affiliations:** 1 Department of Surgical Oncology, Regional Hospital of Jendouba, Jendouba, Tunisia; 2 Faculty of Medicine of Tunis, Tunis Elmanar University, Tunis, Tunisia; 3 Department of Surgical Oncology, Salah Azaïz Institute of Cancer, Tunis, Tunisia; 4 Department of Gastroenterology, Charles Nicole Hospital, Tunis, Tunisia

**Keywords:** Ectopic, Breast, Carcinoma, Management, Case report

## Abstract

**Background:**

Ectopic breast tissue is present in 2–6% of women. Ectopic mammary tissue can experience physiological changes and the same pathological processes as the eutopic breast. Ectopic breast cancer represents an uncommon condition accounting for 0.3% of all breast neoplasms, and it is most frequently located in the axilla.

**Case report:**

We report a rare case of a 57-year-old Tunisian woman who presented with a left-sided axillary mass evolving for about 1 month. The axillary ectopic breast tissue containing the mass was excised with axillary dissection. Pathology revealed a medullary multifocal carcinoma and metastasis was detected in two lymph nodes. She had local radiotherapy after six cycles of chemotherapy. She received herceptin therapy and hormonotherapy. After a 2-year follow-up, no evidence of local recurrence or distant metastases have been identified.

**Conclusion:**

Ectopic breast carcinoma is a rare entity that should be the first diagnosis to be considered if an axillary lump is present in ectopic breast tissue. No particular guidelines on diagnosis and treatment are available. Therefore, physicians should be aware of this condition to avoid treatment delays. Once diagnosed, careful patient follow-up is essential because of the ambiguous natural history of this rare entity.

## Introduction

Ectopic breast tissue (EBT) is present in 2–6% of the population [1]. It might occur anywhere along the thoracoabdominal portion of the milk lines, which stretches anatomically from the axilla to the inguinal area [2]. However, the axilla is the most common presentation site [3, 4]. EBT is susceptible to all physiological and pathological changes that occur in the normal breast, including cancer.

Primary EBC is rare, accounting for just 0.3% of all breast neoplasms [5, 6]. On the other hand, medullary carcinomas represent a minor proportion of these uncommon tumors.

We aimed to shed light on this unusual occurrence. Thus, we report the case of a 57-year-old Tunisian woman who presented with an axillary lump, histopathologically diagnosed as invasive medullary carcinoma arising in EBT.

## Case presentation

A 57-year-old post-menopausal Tunisian woman, non-smoker, multiparous G6P4A2, with a low socioeconomic level presented with a painless left-sided axillary mass evolving for about 1 month. Personal medical and surgical history was unremarkable. At the moment of the clinical examination, she reported no personal or family history of breast, uterine, or ovarian cancer.

Physical examination revealed a 50-mm, firm, well-defined mass in the left axilla. It was very adherent to the skin. The breast examination found no apparent anomaly, and there were no axillary nor supraclavicular nodes. No other abnormalities were seen in the rest of the somatic examination. Results of routine blood examination as well as tumor markers (CA15-3) were within the normal range.

**Fig. 1:**
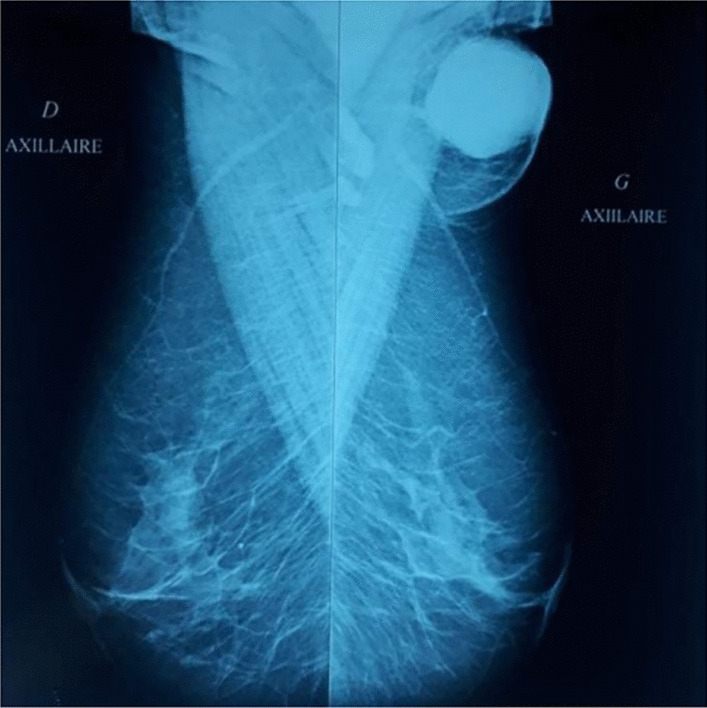
Mediolateral oblique mammography view revealing an ectopic breast with an ill-defined opacity

**Fig. 2:**
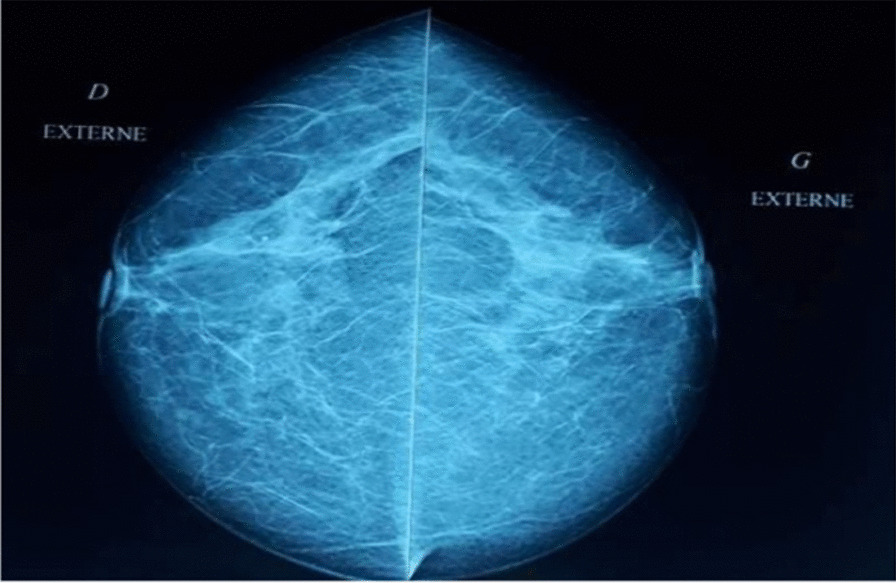
Normal bilateral craniocaudal mammography view

**Fig. 3:**
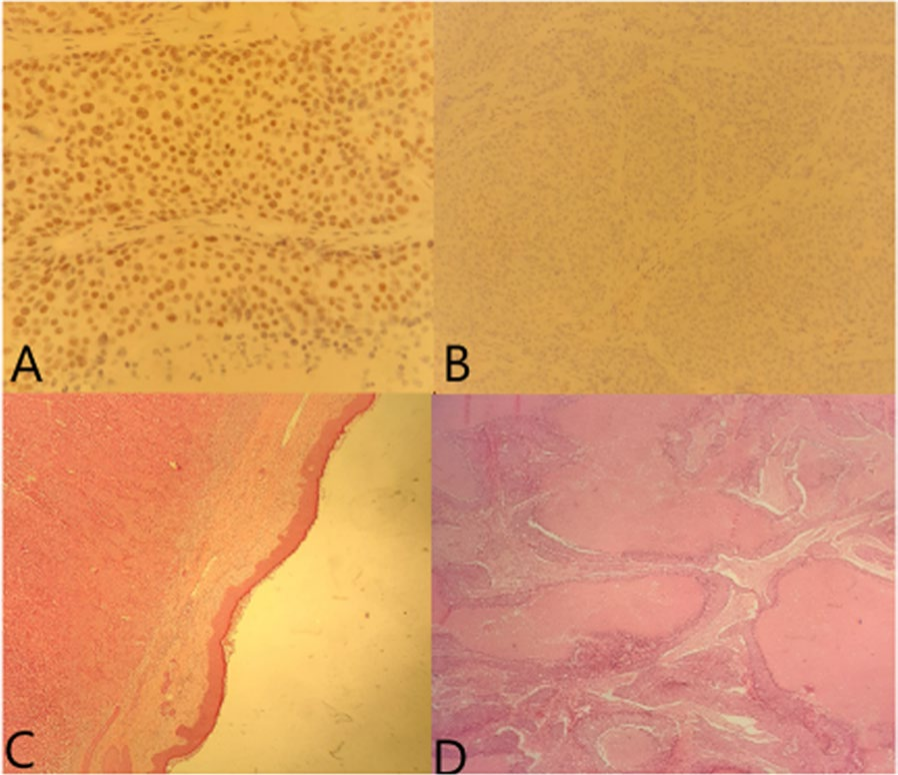
**A** Low estrogen labeling (*400); **B** Absence of labeling for progesterone receptors (*200); **C** Subcutaneous carcinomatous proliferation (*40); **D** Carcinomatous masses with comedonecrosis (*40)

A standard bilateral mammogram was performed and was normal (Fig. 1). This was followed by a dedicated mediolateral oblique mammographic image of the ipsilateral breast (Fig. 2) and an ultrasound of the left axilla, which revealed a solid hypervascular suspicious hyperechoic mass protruding into the skin and measuring 4 cm. Wide resection of the axillary lump was performed. Histopathology concluded with the diagnosis of EBC revealing a medullary multifocal carcinoma with free margins and partial subcutaneous proliferation, positive HER status (score: 3+), low progesterone receptors expression, negative estrogen receptors, and Ki67 score of 80% (Fig. 3).

Enhanced magnetic resonance imaging (MRI) was indicated to eliminate occult breast metastases and showed no other simultaneous lesions. A thoracoabdominopelvic computed tomography (CT) scan was performed and did not reveal any secondary localization. Thus, we conducted an ipsilateral lymph node dissection. There were 2 positive axillary lymph nodes on histologic analysis out of 20.

Then, according to a multidisciplinary meeting decision, she had six cycles of intravenous, systemic, adjuvant chemotherapy based on 5-fluorouracil–epirubicin–cyclophosphamide (FEC) associated with Herceptin, with no severe adverse effects, followed by locoregional radiotherapy. Tamoxifen was also used as endocrine therapy for an additional 5 years following the completion of her chemotherapy. The patient is in good health after a 2-year follow-up, with no evidence of local recurrence or distant metastases.

## Discussion

We reported a rare case of invasive medullary carcinoma arising in EBT. In fact, the prevalence of EBT ranges from 0.4% to 6% in females and from 1% to 3% in males [7]. The axilla, as mentioned in our case, is the most typical location; however, the sternum, infraclavicular region, epigastrium, and vulva have also been described [5, 8]. In up to one-third of patients, EBT might be found in various locations [4].

The ectopic mammary tissue can experience physiological changes associated with menstrual cycle phases, pregnancy, and even the lactation period, much like the breast tissue in its anatomical position [5, 9, 10]. Similarly, the ectopic breast tissue undergoes the same pathological processes as the eutopic breast [6, 9].

Fibroadenomas, fibrocystic alterations, atypical ductal hyperplasia, phyllodes tumors, mastitis, and abscesses have all been reported in ectopic breasts [10, 11].

Although breast cancer is the most prevalent malignancy in women, primary ectopic breast carcinoma (PEBC) is uncommon, accounting for 0.3% of all breast malignancies [5, 8]. Evans *et al*. and Nardello *et al*. reported that PEBC is most commonly seen in the axilla, accounting for 58% to 71% of all cases [12, 13].

Owing to this condition’s low prevalence and misidentification, the average time diagnosis is 40.5 months [12, 14]. These lesions are frequently misdiagnosed and may be challenging to identify from benign (skin tag, nevus, lipoma, hidradenitis) or malignant (nodal metastasis, adnexal tumors) axillary masses [15–17]. PEBC may present as normal-appearing ectopic breast tissue or as an ulcerated lesion [18, 19], similarly to our case. The appearance of a subcutaneous tumor along the mammary line should alert to the likelihood of PEBC, and the presence of suspicious nodules necessitates histologic examination [12, 20].

Preoperative ultrasonography–mammography is a common procedure. In our case, we considered that it is appropriate to perform MRI because, as suggested in the literature, it might be used to rule out a primary ipsilateral occult primary breast cancer [20] or to aid surgical planning by identifying the tumor's size and amount of involvement [14].

The diagnosis of PEBC is confirmed histologically, and ductal carcinoma is described as the common subtype. However, other types of breast cancer, such as lobular, medullary, and papillary carcinomas, have been identified [13]. According to Marshall *et al*., histological types were distributed as follows: 79% of invasive ductal carcinomas, 9.5% of lobular carcinomas, and 9.5% of medullary carcinomas [4].

As reported in our case, medullary carcinoma is a rare and unique subtype of breast carcinoma, accounting for fewer than 5% of all invasive breast malignancies [21].

Despite the lack of published medical literature on PEBC therapy or management guidelines because of the rarity and scarcity of data, orthotopic breast cancer paradigms should be implemented [12, 14].

EBT used to be treated by modified radical mastectomy, excision of ectopic breast tissue, and lymph node dissection; however, patients treated exclusively by excision of the ectopic gland showed encouraging survival rates [5, 13, 16]. Local recurrence can result or occur in both surgical approaches, according to Cogswell *et al*. [22]. As a result, if the breast is free of any malignant lesion, ipsilateral mastectomy, both radical and modified, is no longer recommended [12, 14].

Our patient’s surgical treatment consisted of wide excision and lymph node dissection with no evidence of local recurrence after 2 years of follow-up.

No published studies evaluate the use of the adjuvant treatment in EBC; only individual patient case reports are available. So if there is no concurrent breast tumor, similarly to our case, surgical excision with large margins of the main tumor combined with lymph node dissection [5, 12, 14], followed by radiation therapy, chemotherapy, or endocrine therapy, is then the ideal procedure for a localized stage [10].

Evidence on long-term follow-up data and management of PEBCs is limited and ambiguous [4, 5, 10]. EBC appears to have a worse prognosis than cancer in normal breast parenchyma. The prognosis is thought to be poorer because of the diagnosis delay [10] and the potential to spread regional lymph nodes earlier than typical breast cancer [5, 12, 23, 24].

Owing to the lack of prognostic findings, we believe that a prophylactic excision of ectopic tissue may be indicated for some patients with breast cancer risk factors for whom thorough and close monitoring is difficult [5, 25]. On the other hand, Roorda *et al*. believe that preventive removal of all ectopic breast glands is required since EBC has a poor prognosis [26].

## Conclusions

Ectopic breast carcinoma is a rare entity that should be the first diagnosis to be considered if an axillary lump is present in ectopic breast tissue. Once diagnosed, these patients should follow breast cancer guidelines for staging and therapy. Early-stage patients may have radical excision and axillary lymphadenectomy, as well as adjuvant radiation coupled with endocrine treatment and/or chemotherapy, if indicated. Careful follow-up of these patients is essential because of the ambiguous natural history of this rare entity.

## Data Availability

The datasets used and/or analyzed during the current study are available from the corresponding author on reasonable request.

## Abbreviations

EBT: Ectopic breast tissue
EBC: Ectopic breast cancer
PEBC: Primary ectopic breast cancer
MRI: Magnetic resonance imaging
